# Agricultural dust derived bacterial extracellular vesicle mediated inflammation is attenuated by DHA

**DOI:** 10.1038/s41598-023-29781-9

**Published:** 2023-02-16

**Authors:** Art J. Heires, Derrick Samuelson, Daniel Villageliu, Tara M. Nordgren, Debra J. Romberger

**Affiliations:** 1 VA Nebraska Western Iowa Health Care System, Omaha, NE USA; 2 Department of Internal Medicine, Pulmonary, Critical Care & Sleep division, University of Nebraska Medical Center, Omaha, NE 68198 USA; 3 Department of Environmental and Radiological Health Sciences, Colorado State University, Fort Collins, CO USA

**Keywords:** Inflammation, Immunology, Microbiology

## Abstract

Dietary long-chain omega-3 polyunsaturated fatty acids (n-3 PUFA) and their pro-resolving metabolites are protective against atherosclerotic disease, and ameliorate systemic inflammatory conditions including lupus erythematosus, psoriasis, and bronchial asthma. Organic bioaerosol inhalation is a common and injurious hazard associated with agricultural occupations such as work in swine concentrated animal feeding operations (CAFOs) and is known to increase the risk for developing respiratory conditions such as asthma and COPD. Nearly all cells secrete membrane-bound vesicles (extracellular vesicles, EVs) that have the capacity to transmit protein, nucleic acid, and lipid signaling mediators between cells. Using a polymer-based isolation technique (ExoQuick, PEG) followed by ultracentrifugation, EVs were isolated from CAFO dust extracts, and were quantified and partially characterized. Here, we investigated the role of the n-3 PUFA docosahexaenoic acid (DHA) as a component of n-6 to n-3 PUFA mixtures used to recapitulate physiologically relevant dietary ratios in the resolution of inflammatory injury caused by exposure to EVs carried by agricultural organic dust in vitro. Primary human bronchial epithelial cells, fibroblasts and monocyte-derived macrophages were exposed to EVs isolated from swine CAFO dust. Cells were treated with mixtures of n-6 and n-3 PUFA during recovery from the EV-induced injury. CAFO dust extract (DE) was found to contain EVs that contributed significantly to the overall consequences of exposure to complete DE. DHA-rich PUFA ratios inhibited DE-derived EV-induced proinflammatory cytokine release dose-dependently. DHA-rich PUFA ratios also reversed the damaging effects of EVs on recellularization of lung matrix scaffolds, accelerated wound healing, and stimulated the release of pro-resolution mediators. These results underscore the importance of n-3 PUFA as anti-inflammatory compounds during recovery from EV-laden environmental dust exposure in the context of cellular responses in vitro*,* warranting future translational studies.

## Introduction

Airborne bioaerosols such as those found in modern concentrated animal feeding operations (CAFOs) pose significant health risks to exposed workers including asthma exacerbations, bronchitis, and organic dust toxic syndrome^1^. Dust samples collected from swine and poultry CAFOs have been actively studied and found to contain a complex mixture of feed particles, fecal and soil matter, animal dander, proteases, and bacterial and fungal components^2–5^. Dust samples from swine, poultry and dairy farming operations provoke vigorous inflammatory responses in human^6,7^ and mouse lungs^8,9^, and in lung epithelial^10^ and myeloid cells^11,12^ in vitro. Although it has been suggested that Gram (−) bacterial endotoxins may represent the major dust constituent responsible for the pronounced inflammatory responses to CAFO dust inhalation^4^, other endotoxin-independent factors contained in dust contribute, in part, to DE-mediated inflammatory activities. The observations that CAFO dust scrubbed of its endotoxin content was still able to elicit inflammatory responses in epithelial cells and monocytes^13^, and toll-like receptor 2 (TLR2) activation in a dust exposure mouse model indicate involvement of other signaling factors including Gram (+) bacterial components^14^. It remains unclear which and to what extent the myriad constituents of agricultural dust are responsible for its potent inflammatory properties.

Extracellular vesicles (EVs) are heterogenous membrane-bound structures shed by endocytosis or by budding from the plasma membrane of most cells including fungal, bacterial, mammalian, and plant cells^15,16^. EVs are generally defined as vesicles ranging in size from 100 to 1000 nm in diameter. Once thought to function as vehicles for cellular waste disposal^17^, EVs are now recognized as a means of intercellular communication, allowing cells to exchange messages via their nucleic acid, protein, and lipid cargos. EVs are known to be involved in both normal physiological functions and alarm signaling during pathological processes by transporting bioactive molecules to both nearby and distant cells^18,19^. The cargo transported by EVs, their release and abundance can be influenced by external stimuli including environmental injury; for example, signals packaged in EVs originating from stressed cells can transduce alert signals to bystander cells^20^. Swine CAFO dust contains bacterial components and is therefore likely to carry bacterial-derived EVs (outer membrane vesicles or OMVs), and potentially swine-, fungal-, and human-derived EVs. The signaling moieties transmitted by these diverse sources are largely unknown, although a recent article demonstrates the presence of bacterial EVs in poultry CAFO dust and examines their inflammatory effects^13^. In this study, we sought to identify and partially characterize EVs carried by swine CAFO dust, and to evaluate their contribution to the potent inflammatory effects of CAFO dust exposure.

Dietary long-chain polyunsaturated acids (PUFA) have been shown to exhibit significant anti-inflammatory properties in numerous studies^21,22^. Two classes of PUFA are the omega-6 (n-6) and omega-3 (n-3) PUFA. Both are structural components of the phospholipid bilayer in eukaryotic cell membranes; they are considered essential fatty acids because they cannot be synthesized de novo in humans. The n-6 PUFA arachidonic acid is known to mediate inflammatory activation by formation of eicosanoids such as leukotrienes, prostaglandins, and thromboxanes^23^. High levels of dietary n-6 PUFA typical of Western diets is associated with many debilitating afflictions including cardiovascular, autoimmune, and inflammatory diseases^24,25^. Conversely, dietary n-3 PUFA such as eicosapentaenoic acid (EPA) and docosahexaenoic acid (DHA) are known to have anti-inflammatory or pro-resolving properties and are the major constituents of fish oil dietary supplements. Omega-3 PUFA act as substrates for the synthesis of specialized pro-resolving mediators (SPM) such as resolvins, maresins, and protectins, as well as endocannabinoids, all of which generate anti-inflammatory effects. A healthy balance of dietary PUFA (n-6:n-3 ratio; termed the “omega-3 index”^26^, can contribute to the reduction of inflammation in many chronic conditions. It has been suggested that a 1:1 ratio of n-6:n-3 PUFA would be ideal, but Western diets are woefully deficient in n-3 PUFA and include excessive n-6 PUFA; averaging 16:1, with values as high as 40:1 being reported. This dietary imbalance is due to the post-agricultural shift toward increased consumption of cereal grains and hydrogenated vegetable oils (sources of n-6 PUFA) and away from fatty fish, nuts, and seeds^27^. Compelling therapeutic effects of n-3 PUFA supplementation have been demonstrated for chronic inflammatory conditions such as rheumatoid arthritis^28^, ulcerative colitis^29^, lupus erythematosus^30^, atherosclerosis^31^, and asthma^32^. A recent clinical study demonstrated the beneficial effects of diets enriched for n-3 PUFA on lung function in individuals exposed to air pollution^33^. The authors emphasized the importance of a healthy n-6:n-3 PUFA ratio for the protective effects of dietary intervention. We have previously shown that a DHA-enriched diet remediates the adverse effects of environmental dust exposure in mice^34^ and exogenous DHA mitigates dust-induced lung cell inflammation in vitro^35,36^.

In this study, to further explore the role of EVs from agricultural environments in eliciting lung inflammation, and assess the impacts of a low n-6:n-3 PUFA ratio in mitigating these inflammatory effects, we have utilized primary human lung cell cultures, monocyte-derived macrophages, and an ex vivo lung scaffolding model of regeneration to assess the relationships between agricultural dust-derived EVs and cellular inflammation responses. Our results demonstrated that swine CAFO dust extract (DE) is replete with EVs containing bacterial and viral DNA and that DE-derived EVs generate potent inflammatory responses in recipient cells. In addition, we show that treatment with a PUFA ratio enhanced for n-3 (DHA) attenuated the injurious effects of proinflammatory alarm signals transported by DE-derived EVs in vitro, with an emphasis on the resolution phase of the inflammatory response.

## Results

### CAFO dust extracts contain extracellular vesicles

**Figure 1:**
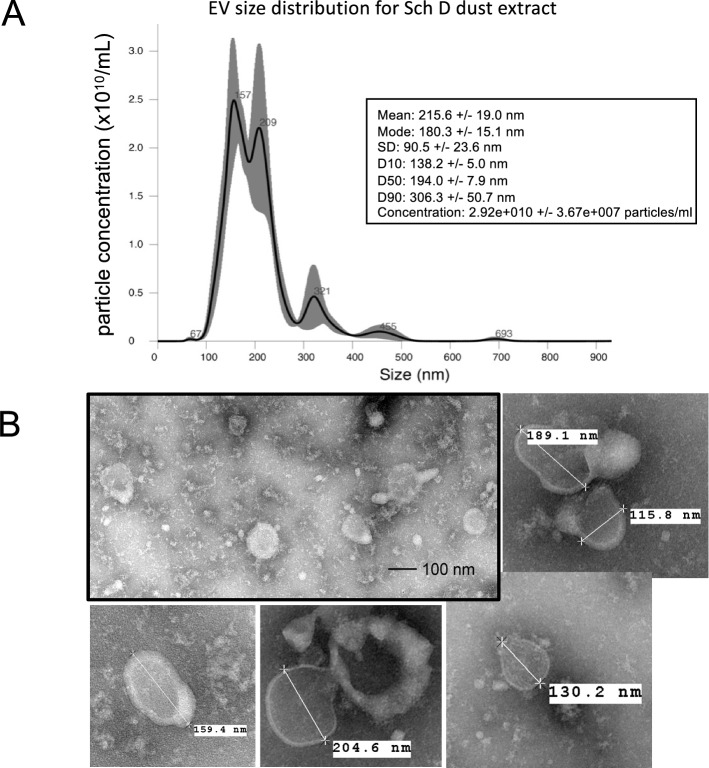
EVs are abundant in DE-derived dust samples. Nanoparticle tracking analysis of 100% DE-derived EVs indicated a concentration range of 2.5–3.8 × 10^10^ particles/mL and a mean size of 215 nm (**A**). Representative transmission electron micrographs illustrate the concentration and size distribution of EV preps (**B**). In all subsequent experiments the Sch D DE-derived EVs were used.

Nanoparticle tracking analysis (NTA) of DE-derived EVs from dust samples collected from swine confinement facilities demonstrated that DE contains an abundance of EVs (2.5–3.8 × 10^10^ particles per mL (Fig. 1A). Mean EV concentration in 100% DE (Sch D sample) was 2.8 × 10^10^ particles/mL which corresponds to 1.46 × 10^9^ particles/mL in 5% DE or 2.5 × 10^8^ EV per µg protein in the EV pellet (Fig. 4D). Representative transmission electron micrographs of a typical EV prep demonstrate the abundance and relative size homogeneity of EVs isolated from DE (Fig. 1B). Dust extract-derived EVs exhibit a mean particle size of approximately 215 nm (mode: 180.3 nm, by NTA, with the majority of particles below 250 nm). Although a subset of the particles are in the size range of exosomes (≤ 100 nm), for precision, the particles are referred to in this text as EVs.

### Dust-derived EVs contain bacterial and viral DNA

**Figure 2:**
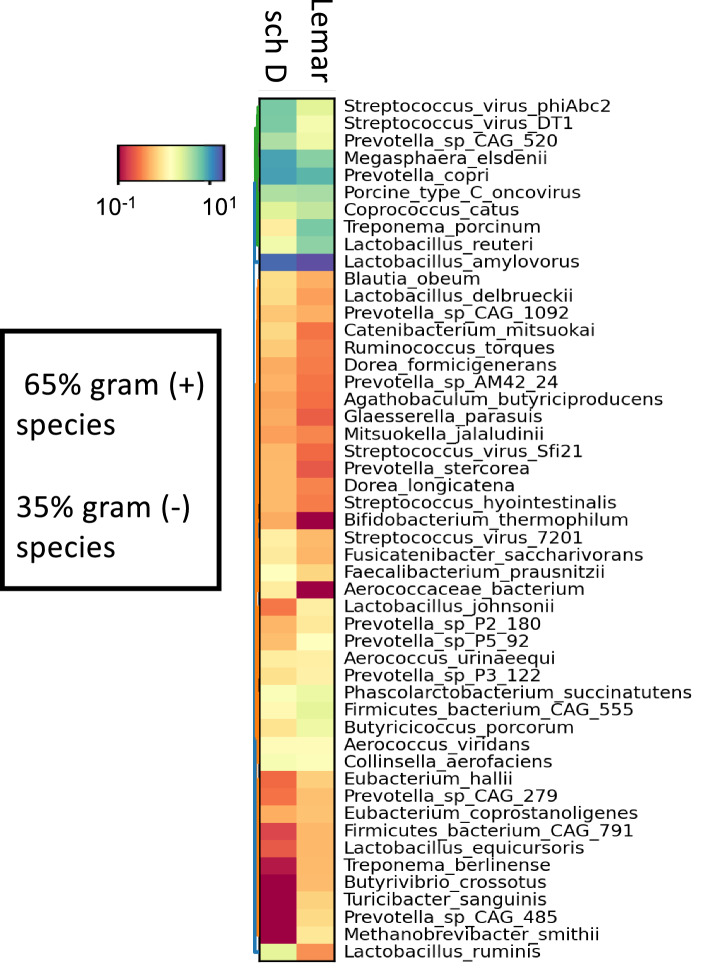
Dust-derived EVs contain DNA from both Gram (+) and Gram (−) bacterial species, as well as viral (bacteriophage) signatures. The 50 most prevalent signatures originated predominantly from Gram (+) bacteria (65%) commonly found in feces, soil, and as part of the normal swine gut flora. Several pathologic species were also identified.

Genotyping of dust EV DNAs isolated from two different dust samples collected from two different swine CAFOs was performed by shotgun pyrosequencing metagenomic analysis. The taxonomic profiles derived from whole genome sequence data indicated that the EVs were derived from Gram (+) and Gram (−) bacteria, several bacteriophage species, and pathogenic swine microbes (Fig. 2). The preponderance of bacterial species identified in the 50 most abundant signatures were Gram (+) (65%), and were comprised largely of gut, fecal and soil organisms. Mammalian and human DNA signatures were not detectable among the 50 most abundant species. Taxonomic data also showed that the different dust EV samples shared similar bacterial signatures but with varying frequencies.

### DE-derived EVs induce potent inflammatory mediator responses in bronchial epithelial cells

**Figure 3:**
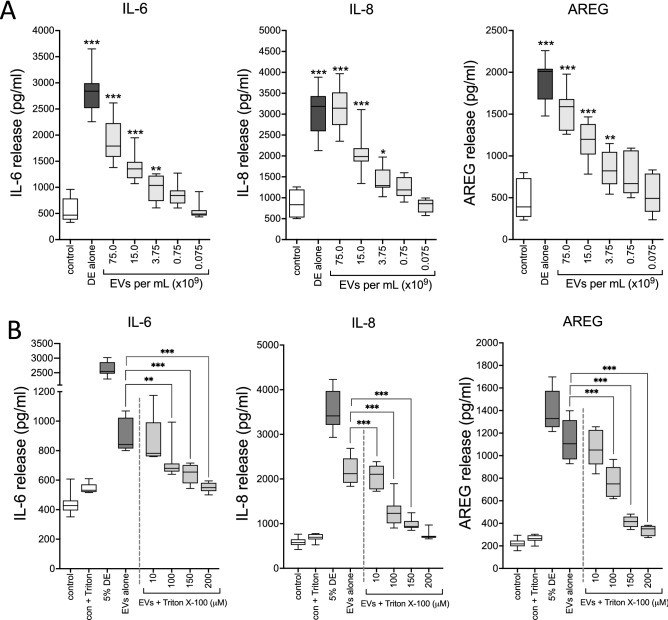
DE-derived EVs dose-dependently stimulated inflammatory mediator release from bronchial epithelial cells, and detergent lysis of EVs reduced their potency. BEAS-2B cells were treated with various concentrations of DE-derived EVs, or 5% complete DE, and incubated for 24 h. IL-6, IL-8, and AREG were measured in culture supernates (**A**). Soluble protein release from HBEC challenged with EVs recovered from DE treated with doses of Triton X-100 is shown in (**B**). The medians ± quartiles of 3 independent experiments are shown (n = 12 technical replicates per condition for (**A**) and 8 replicates for (**B**). *p < 0.05, **p < 0.01, ***p < 0.0001 vs control (or for indicated comparisons in (**B**) (ANOVA, Tukey’s post-hoc test).

To assess the capacity of DE-derived EVs to elicit inflammatory mediator release, immortalized epithelial cells (BEAS-2B) were exposed to various concentrations of EVs for 24 h, and release of soluble mediators was measured by ELISA. IL-6, IL-8 and AREG release were prominently and concentration-dependently elevated following DE-derived EV exposure, with even low concentrations eliciting significant upregulation (Fig. 3). Although the highest dose of EVs was more concentrated than the EVs found in complete 5% DE (~ 1.46 × 10^9^ EV/mL), these results demonstrate the capacity of DE-derived EVs to generate robust inflammatory responses in epithelial cells.

**Figure 4:**
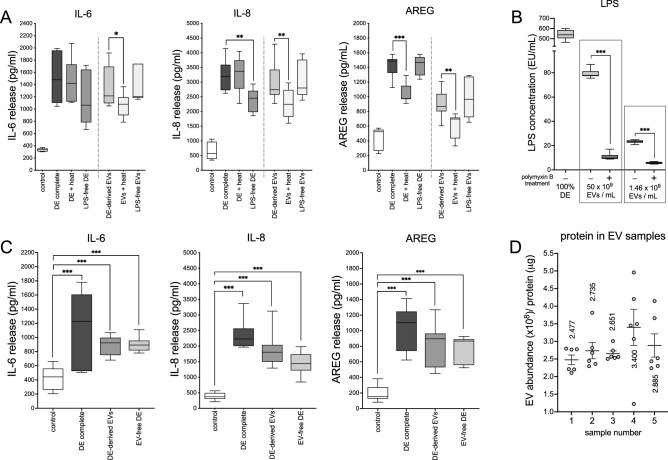
Stimulatory effects of EVs are sensitive to heating but are not due to LPS contamination and the effects of complete DE are partially due to the EV cargo. Saturated (100%) DE was subjected to heat (90 °C for 15 min) or LPS ablation (polymyxin B, 12 h @ 4 °C). HBEC were challenged with EVs isolated from these preparations (5% DE equivalents) or with heat-or polymyxin B-treated 5% DE for 24 h (**A**). Polymyxin B treatment significantly depleted LPS from EV suspensions (**B**). Alternatively, cells were challenged with complete 5% DE, an equivalent concentration of DE-derived EVs, or DE devoid of EVs, for 24 h. Supernatant medium was assessed for IL-6, IL-8 and AREG levels by ELISA (**C**). EV abundance (NTA) as a proportion of total protein for 5 different preps is shown in (**D**). Data shown for (**A**) and (**C**) are means of 3 independent experiments (8 or 14 technical replicates per condition). *p < 0.05, **p < 0.01, ***p < 0.0001 vs control (AVOVA, Tukey’s post-hoc test). For (**B**) and (**D**), 6 technical replicates per condition, ***p < 0.001, Student’s *t* test.

To further characterize the immunomodulatory properties of DE-derived EVs, complete 100% DE was subjected to heat (90 °C, 30 min) or LPS ablation (polymyxin B resin, 4 °C, 12 h), and EVs were isolated. Heat- and polymyxin B-treated complete DE and DE-derived EVs were then used to stimulate HBEC cultures for 24 h. Heat had no effect on DE-induced IL-6 and IL-8 release but did reduce AREG release. Polymyxin B treatment effectively removed 75–88% of measurable LPS from isolated EVs (Fig. 4B). Ablation of LPS from complete DE significantly attenuated IL-8, and modestly decreased IL-6 release, but had no effect on AREG. When DE-derived EVs were exposed to high heat, release of all three mediators was inhibited, but removal of LPS had no effect on EV-induced release for any mediator (Fig. 4A). Thus, DE-derived EVs contain heat-sensitive constituents, but their effects are not attributed to LPS which is an integral Gram (−) bacterial outer membrane vesicle component. EVs also contribute to the immunomodulatory effects of DE; fully half of the inflammatory capacity of complete DE is due to EV cargo. Complete 5% DE was fractionated into DE-derived EVs and EV-free DE (soluble components) and primary HBEC were treated with the fractions. The EV component was responsible for nearly 50% of the immunomodulating effects of complete DE, although the responses generated by the two components were not precisely additive (Fig. 4B). This was anticipated because EV-free DE and DE-derived EVs undoubtably signal through shared pathways. These results demonstrate the essential difference between complete DE containing soluble inflammatory components such as LPS, and the vesicular fraction which acts through its myriad cargo of intercellular signaling factors. To assess the efficacy of the PEG-based precipitation method for bioactive EV isolation, validation experiments were performed using the more traditional differential centrifugation (DC) technique described in “Methods”. Results included as supplemental material (Figs. S1, S2) verify the two methods are comparable in terms of in vitro modulator release.

### DHA-rich PUFA ratios reversed the deleterious effects of DE-derived EV exposure on recellularization of lung scaffold cultures

**Figure 5:**
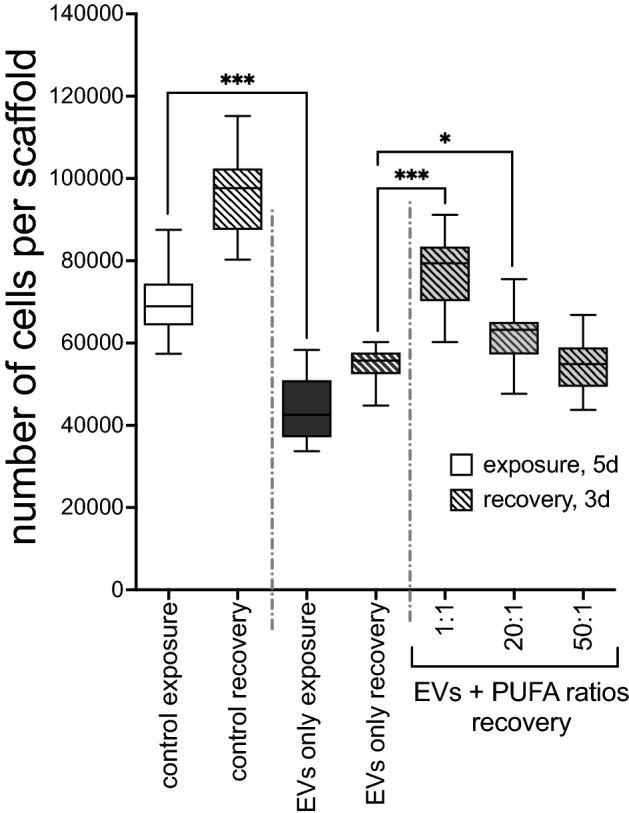
DHA-rich PUFA ratios restored the EV-induced suppression of recellularization on lung matrix scaffolds. Human lung scaffolds were seeded with HBEC, challenged with 50 × 10^9^ EVs/mL for 5d (“exposure,” solid bars) then allowed to recover in the presence of PUFA ratios for an additional 3d (“recovery,” hatched bars). DE-derived EVs hindered the repopulation of scaffolds during both exposure and recovery, while DHA-rich mixtures reversed the recellularization deficit. *p < 0.05, ***p < 0.0001 (ANOVA, Tukey’s post-test, n = 3 experiments, 12 or 16 technical replicates per condition).

With increasing interest in dietary n-3 PUFA as anti-inflammatory agents, and especially the balance between n-6 and n-3 PUFA^26,31^, we designed experiments using various equimolar ratios of AA (n-6) and DHA (n-3) representing a favorable DHA-rich 1:1 ratio, and unhealthy AA-rich ratios (20:1 and 50:1) to determine their effects on DE-derived EV-mediated disruption of injury repair and regeneration. To this end, we employed a novel 3-dimensional human lung scaffold recellularization model. Denuded lung matrix scaffolds were seeded with HBEC and exposed to 50 × 10^9^ EVs/mL for 5d (exposure phase). Partially repopulated scaffolds were then rinsed, and EVs removed prior to being treated with the PUFA mixtures or control medium for an additional 3d (recovery phase). The rate of recellularization on the control scaffolds continued to increase during recovery, but the EV-exposed cells demonstrated a reduced ability to repopulate the scaffolds, indicating a residual adverse effect even after EVs were removed. The DHA-rich (1:1) PUFA ratio partially reversed the potent injurious effects of EV challenge during the 3d recovery period (43.4% improvement in recellularization vs. EVs only) while the 20:1 mixture had a modest effect, and the 50:1 ratio had no effect (Fig. 5). Recellularization of scaffold cultures treated with EVs followed by 2 mM DHA alone was no different than the 1:1 PUFA ratio (not shown). These results demonstrate that exposure to DE-derived EVs induced a marked repair deficiency in the epithelial cells, and that DHA-rich PUFA ratios aided restoration of normative resolution.

### DHA-rich PUFA ratios remediated the impaired wound-healing capacity of EV-exposed lung cells

**Figure 6:**
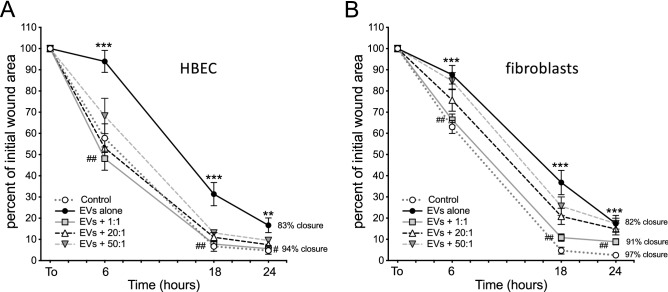
EV-induced wound resolution deficit was reversed in the presence of DHA-rich PUFA ratios. Circular wounds made in confluent monolayers of HBEC (**A**) or HLF (**B**) were allowed to heal in the presence of control medium, DE-derived EVs, or EVs with one of three PUFA ratios. The wound area was analyzed over the course of 24 h, compared to the size of the initial wound, and percent closure was calculated. DHA-rich PUFA ratios (1:1) significantly reversed the DE-mediated wound closure deficit. Results shown are means for 3 independent experiments, 12 (**A**) or 9 (**B**) technical replicates per condition. **p < 0.01, ***p < 0.0001 vs. Control, ^##^p < 0.001 vs. EVs alone at corresponding time points, (Student’s *t* test for pairwise comparisons).

As an alternate model for the analysis of PUFA ratios on wound resolution, we employed a classical scratch-wound assay. Circular wounds were made in confluent monolayers of primary HBEC or HLF, and the progress of wound repair was monitored in the presence of DE-derived EVs alone, and EVs + treatment with the three different PUFA ratios (as above). Under control conditions, wounds were at least 94% closed by 24 h for both cell lines. EV treatment impaired the wound closure rate (12% and 16% inhibition of closure for HBEC and HLF respectively, at 24 h). When monolayers were concurrently exposed to EVs and PUFA ratios, the repair deficit was reversed DHA-dependently with the DHA-rich (1:1) ratio markedly attenuating the impairment as early as 6 h approximating the wound closure rate of control wells (Fig. 6). The effect of the 1:1 PUFA ratio was equivalent to treatment with 2 mM DHA alone (not shown for clarity). The 20:1 and 50:1 AA:DHA ratio doses had intermediate effects on EV-induced repair deficits. These results further validated the effectiveness of DHA-rich PUFA treatment in the resolution of dust-derived EV-induced injury.

### DHA enriched PUFA ratios inhibited EV-induced inflammatory cytokine release and enhanced AREG release during recovery

**Figure 7:**
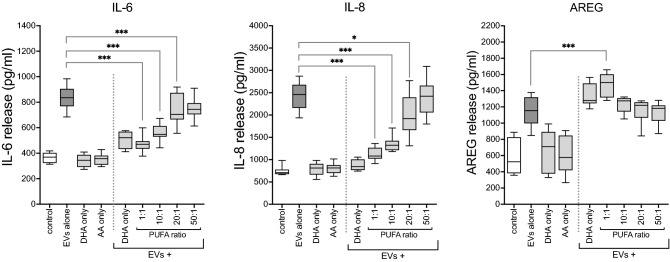
DHA-rich PUFA ratios dampened DE-derived EV-induced inflammatory cytokine release, but augmented AREG release during recovery. HBEC were stimulated with concentrated DE-derived EVs (50 × 10^9^/mL) for 2 h, EVs were then removed, and cultures were allowed to recover in the presence of PUFA ratios for 22 h. Release of EV-mediated proinflammatory cytokines IL-6 and IL-8 was attenuated, and pro-repair AREG was augmented by treatment with the DHA-rich (1:1) PUFA ratio and by DHA alone during the recovery phase. Data shown are means of 4 independent experiments with 24 technical replicates per condition. *p < 0.05, **p < 0.01, ***p < 0.0001 (ANOVA, Tukey’s posttest).

Because the bronchial epithelium functions to mediate lung inflammatory and immune processes^37^, and n-3 PUFA are known to modulate these functions in the context of exposure to environmental irritants^36^, we sought to investigate the effect of exogenous DHA-rich PUFA ratios on EV-mediated inflammatory cytokines, and on the pro-repair mediator AREG. Primary HBEC were challenged with 50 × 10^9^ EVs/mL for 2 h and then allowed to recover in the presence of various mixtures of PUFA or with DHA alone for 22 h. Exposure to EVs alone elevated the release of IL-6, IL-8 and AREG. Treatment with DHA-rich PUFA mixtures dose-dependently blunted the release of inflammatory cytokines but amplified the release of AREG during recovery from the initial insult (Fig. 7). The highest concentration of DHA (1:1 ratio) nearly abolished the inflammatory effects of EV exposure (IL-6, IL-8), while the high n-6 mixture (50:1) had no effect. The effects of treatment with EVs + DHA alone closely resembled the effects of the 1:1 treatment. Neither DHA nor AA alone had noticeable effects on baseline release of any mediator. These results suggest that dust-derived EV-mediated lung cell inflammatory processes persist following an acute exposure, and that during recovery, n-3-rich mixtures of PUFA attenuate the adverse response. Also, the pro-resolution agent AREG is augmented by treatment with high concentrations of DHA, indicating that dietary n-3 PUFA are not only protective, but are important in the remediation of lung injury.

### DHA-rich PUFA ratios also dampened release of EV-induced proinflammatory cytokines and enhanced pro-resolution mediators during recovery in monocyte-derived macrophages (MDM)

**Figure 8:**
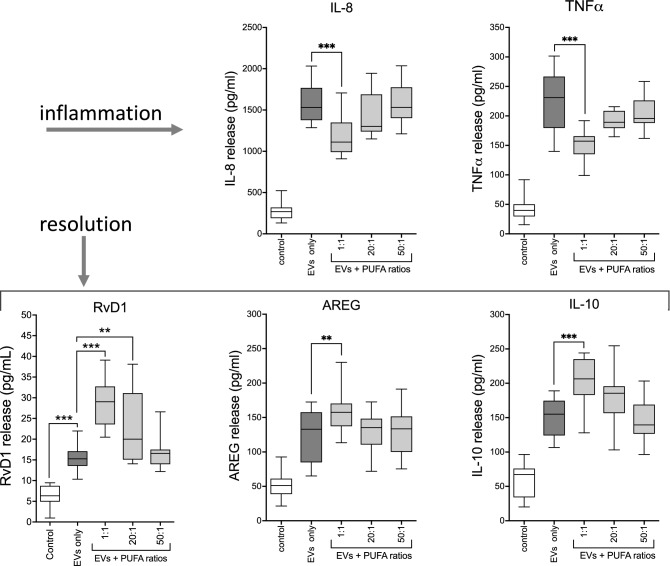
The DHA-rich PUFA ratio (1:1) inhibited EV-mediated proinflammatory cytokine release but augmented pro-repair mediator release from MDM during recovery. Sub-confluent monocyte-derived macrophages were challenged with EVs for 2 h, then allowed to recover in the presence of PUFA ratios for 22 h. The DHA-rich ratio dampened the EV-induced release of inflammatory cytokines (IL-8, TNFα) and elevated the release of pro-repair mediators RvD1, AREG, IL-10. Data pooled from n = 5 experiments, 24 technical replicates. **p < 0.01, ***p < 0.0001, for indicated comparisons (ANOVA, Tukey’s post-hoc test).

Following a 2 h exposure to 50 × 10^9^ EVs/mL, sub-confluent adherent MDM were rinsed, and allowed to recover in the presence of various PUFA ratios for 22 h. EV treatment stimulated a striking increase in the release of all four mediators (55–84% increase compared to control) even after the stimulus was removed. IL-8 and TNFα release was significantly inhibited in the presence of the DHA-rich PUFA ratio while the pro-resolution mediators RvD1, AREG and IL-10 were augmented (Fig. 8). Again, these observations support the conclusion that EVs borne by environmental dusts elicit detrimental inflammatory signals, and that supplementation achieving a PUFA ratio enriched for DHA can mitigate these responses. These results substantiated the epithelial cell observations in an alternate cell type.

## Discussion

Regulation of lung inflammation and injury following occupational exposure to inhaled irritants, such as those encountered in dusty agricultural environments is the focus of ongoing studies. Specifically, it is poorly understood why some individuals fully recover from inhalant injury, while in others, chronic inflammatory lung pathology develops. Characterization of the many potentially inflammatory constituents of environmental dust is likewise incomplete. Findings presented here demonstrate that agricultural dust carries EVs derived from many sources, that dust-borne EVs impart inflammatory signals to recipient cells, and that supplementation with PUFA mixtures rich in n-3 PUFA modulate the exaggerated inflammatory responses during recovery from dust-derived EV exposure.

Microvesicle and exosome research escalated in the mid 1980s after several published studies reported the transfer of labeled proteins to naïve cells by means of exocytosis^38^. Since then, mechanisms by which EVs are formed, loaded, transported, released, and delivered have been thoroughly elucidated^16^. Hundreds of thousands of protein, lipid, and RNA species have been identified in EVs so far. Although initially thought to function in cellular waste removal, more recent evidence indicates that EVs play an essential role in intercellular communication by shuttling both normal physiological and alarm signals to near and distant recipient cells. For example, the response to LPS-induced inflammation was modulated by vesicle-mediated miRNA transfer in dendritic cells^39^, and proinflammatory cytokines were enriched in exosomes isolated from septic mice during early sepsis, while pro-repair cytokines were elevated during late sepsis^40^. Analysis of swine CAFO dust samples indicated that these environmental aerosols are highly complex mixtures comprising Gram (+) and Gram (−) bacterial components, muramic acid, LPS, peptidoglycan, fungal-derived ergosterol, and metals^4^. The constituents of dust-derived EVs, however, are largely unknown. Given this, recent studies have reported adverse inflammatory consequences of exposure to EVs derived from feces^41^, house dust^42^, fungi^43^, and poultry dust^22^. Although we have previously observed that LPS is responsible for some of the injurious effects of complete CAFO dust extract, here we show that EV preparations treated with polymyxin B to eradicate LPS are still able to elicit inflammation in epithelial cells. This would suggest that inflammatory effects of EV treatment are not due to LPS. Indeed, others have proposed that LPS is not a major factor in inflammatory consequences of agricultural dust-derived EV exposure, but stipulate that some endotoxin species are variously sensitive to polymyxin B treatment^13^. We acknowledge the possibility that LPS as a constituent of Gram (−) bacterial outer membranes may contribute to the inflammatory effects we have observed. However, our basic thesis that the EVs carried by environmental dust—including their surface constituents—can have harmful inflammatory effects, remains relevant. The high concentration of DE-derived EVs we chose for the induction of inflammatory responses may not be analogous to a single exposure to airborne agricultural dust, however EVs deposited in the lung during repeated and prolonged environmental dust exposures may be significant, and thus the doses we investigated are likely to be physiologically relevant in the context of cumulative exposure. Studies examining the deposition and persistence of inhaled EVs in the lung are warranted. Bacterial EVs are known to activate NFκB, generating inflammatory cytokine expression. Although the specific mechanisms responsible for this process are largely unknown, some evidence implicates roles for MMP-9, IL-17A, and TLR2 in EVs isolated from household dust^42^. In the present study, dust-derived EVs were found to contain DNA signatures of bacterial and viral species. The taxonomic characterization of these nucleic acid signatures illustrated the complex nature of agricultural dust samples: numerous and diverse organisms contribute to the store of potential signaling moieties encapsulated in dust EVs. Predictably, the predominant contributions to the EV cargo were by soil, fecal, and gut flora organisms, especially Gram (+) bacteria. Somewhat more surprising, however, was the presence of several viral and swine pathogen signatures. Extracellular signaling molecules transported by EVs including proteins and RNA are protected from proteolytic digestion and degradation by means of their membrane-bound structure. In addition, their biocompatibility with recipient cell membranes makes EVs potentially more effective as a means of intercellular signaling than factors secreted directly into the extracellular space^44^. EV cargos may also be protected from degradation after internalization by recipient cells, allowing the efficient activation of pathological or physiological responses.

**Figure 9:**
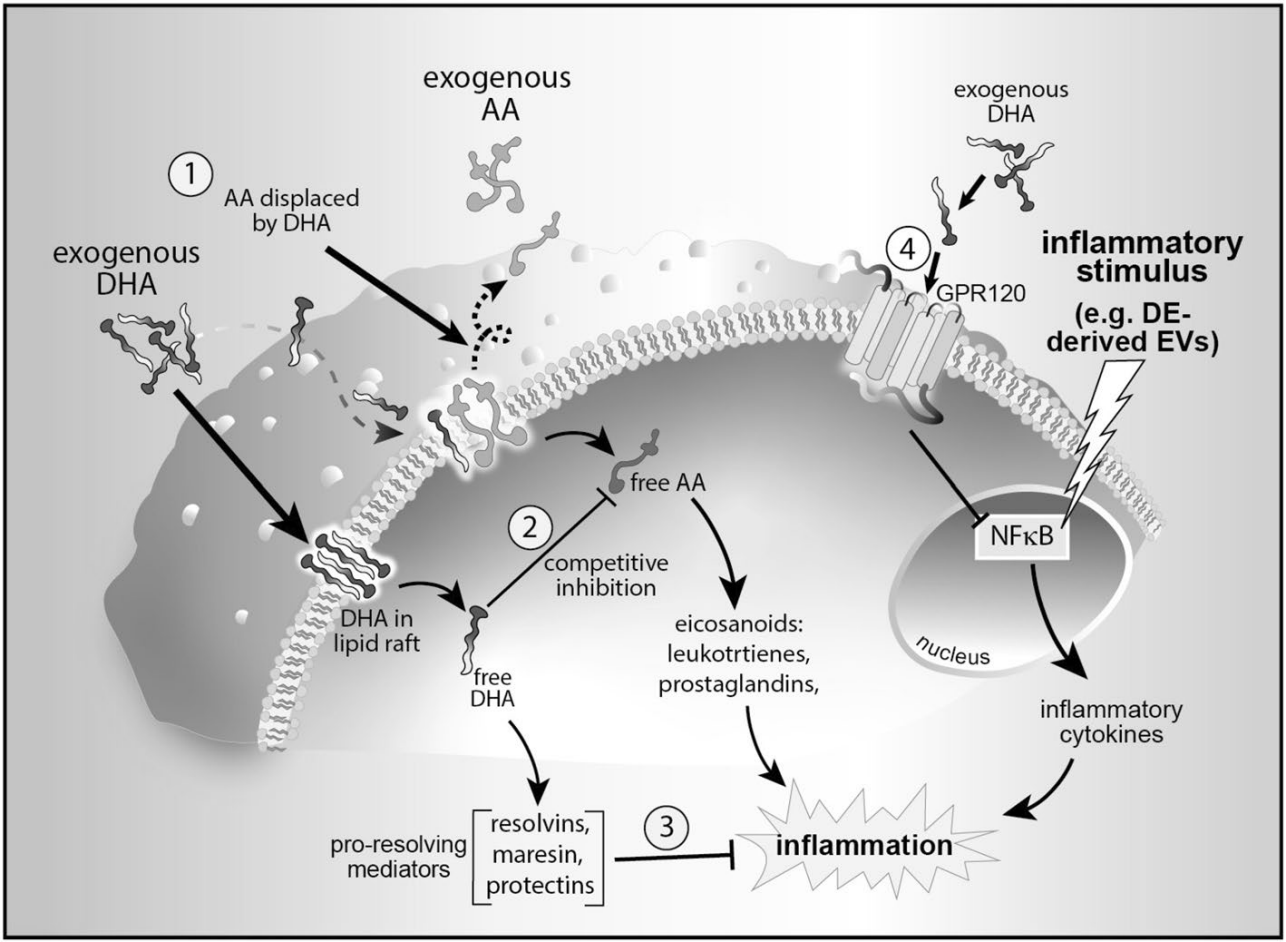
Schematic overview. Mechanism of n-3 PUFA-modulated suppression of EV-induced inflammation. Exogenous DHA competes with the more proinflammatory n-6 PUFA arachidonic acid (AA) for incorporation into cell membranes (**1**). Inside the cell, free DHA inhibits enzymatic conversion of AA to eicosanoids by direct competition (**2**). Specialized pro-resolving mediators are synthesized from free cytoplasmic DHA (**3**). Binding of exogenous DHA to the GPR120 surface receptor interrupts NFκB-mediated inflammatory cytokines (**4**).

Two major classes of PUFA are termed omega-6 (n-6) and omega-3 (n-3) PUFA, based on the positions of the double bonds in the carbon backbone. Both can be incorporated into the phospholipid bilayer of cell membranes. The n-6 PUFA arachidonic acid (AA) is released from membranes upon stimulation and is metabolized to form proinflammatory mediators such as leukotrienes and prostaglandins. Conversely, the n-3 PUFA DHA can displace AA in membranes, thereby reducing its availability, and compete with AA for COX and LOX enzymes for conversion to bioactive metabolites^45^. More importantly, DHA is a substrate for the synthesis of specialized pro-resolving mediators (SPM) such as resolvins, maresins and protectins, (see Fig. 9) which have been shown to mitigate the development of atherosclerotic disease^46^, pulmonary inflammation^47^ and recently in suppression of COVID-19 inflammatory symptoms^48^. The literature is replete with reports supporting the virtues of the n-3 enriched Mediterranean diet for many chronic conditions including cancer, cardiovascular disease, colitis, and diabetes^49^. Many of these studies present evidence substantiating the benefit of a low ratio of dietary n-6 to n-3 PUFA. The use of n-3 PUFA as a countermeasure for diverse chronic inflammatory conditions is well documented. Several studies have investigated the incorporation of DHA in lung and other tissues following treatment with n-3 PUFA-supplemented diets. Increased deposition of DHA in mouse lung^50^, and skeletal muscle^51^, were observed after DHA-supplemented dietary regimens, and an early study demonstrated elevated n-3 PUFA in heart, liver and kidney of marmoset monkeys fed a diet enriched for n-3 PUFA^52^. These studies confirm the translocation of intact n-3 PUFA from the gut to remote tissues following dietary intervention. Beneficial effects of therapeutic doses of DHA have been published for alleviating cachexia and improving quality of life in cancer patients^53^, slowing muscle wasting in aging persons^42^, reducing mortality in pancreatitis^54^, and even improving cognitive function in Alzheimer’s disease^55^. The defective resolution of persistent inflammation in cystic fibrosis patients has been partially attributed to deficient production of SPM^56^. We have previously reported that mice fed a DHA-supplemented diet for 4 weeks demonstrated significantly reduced lung inflammation and inflammatory cytokine production during recovery from repetitive dust extract challenge compared to control-fed mice^34^. We have also reported that DHA treatment attenuated the adverse effects of dust exposure in human bronchial epithelial cells and amplified the release of the pro-repair mediator AREG^36^. These findings illustrate an essential role for n-3 PUFA such as DHA in the mitigation of a dysregulated immune response to an injurious inflammatory environment.

Amphiregulin (AREG), a membrane-anchored EGF receptor ligand, was originally thought to be produced exclusively by epithelial cells and fibroblasts, but is now known to be expressed by many activated immune cells in numerous inflammatory conditions. Accumulating evidence has established a complex role for AREG as a modulator of tissue homeostasis, resistance to infection, tissue remodeling, and resolution of inflammation^57^. In a study comparing sex differences in the pathogenesis of influenza A infection, increased production of AREG resulted in decreased inflammation, and accelerated recovery following viral challenge^58^. In an animal model of induced intestinal inflammation, exogenous AREG treatment ameliorated disease severity and promoted favorable tissue remodeling through induction of IL-33-activated ILC2 cells^59^. Likewise, AREG treatment suppressed the injurious effects of bleomycin-induced lung damage (a model of idiopathic pulmonary fibrosis) through the activation of survival signals^60^. All of these studies emphasize the effectiveness of AREG during the resolution of inflammatory injury, especially when exaggerated or inappropriate immune responses exacerbate disease severity.

In this study we have demonstrated the existence of EVs carried by swine CAFO dust and provided evidence for the promotion of lung inflammatory responses by these abundant dust-borne EVs (Fig. 3). The exceedingly small size of EV particles suggests that deposition and retention of these nanovesicles in distal lung compartments might provoke significant deleterious responses to bioaerosol exposure. Dust-derived EVs were found to contain DNA signatures originating from numerous and diverse sources, with a preponderance (> 65%) of nucleic acids derived from Gram (+) bacteria, although viral signatures were also present. EVs isolated from swine CAFO dust strikingly stimulated proinflammatory cytokine release from bronchial epithelial cells, but also induced the pro-repair molecule AREG. These effects were shown to be independent of LPS-mediated effects (Fig. 4). These observations support our previously published conclusions indicating a beneficial role for AREG in remediating defective repair mechanisms following agricultural dust extract exposure^61^. In the present study, cells were treated with various ratios of arachidonic acid (n-6 PUFA) and DHA (n-3 PUFA) representing a healthy diet, (1:1 ratio), average Western diet, (20:1), and excessive n-6 PUFA-containing diet (50:1), respectively, to more closely approximate real-world PUFA consumption patterns. DHA-rich PUFA ratios stimulated AREG release from HBEC during recovery from an initial acute exposure to EVs and reversed the recellularization deficit induced by EV exposure in a tissue repair model using acellular lung matrix scaffolds (Fig. 5). In complimentary experiments, DHA-rich PUFA ratios mitigated the DE-induced wound repair defect in a classical scratch/wound assay in both HBEC and HLF cultures (Fig. 6). These observations support the potent pro-repair function of n-3 PUFA during recovery from injury. DE-derived EVs triggered proinflammatory cytokine release by HBEC and MDM (IL-6, IL-8, and IL-8, TNFα, respectively) even during recovery. DHA-rich PUFA ratios attenuated this effect, but importantly, also amplified the release of pro-resolution mediators: AREG release was enhanced in HBEC, and RvD1, AREG, and IL-10 were augmented in MDM (Figs. 7 and 8). One consequential observation stemming from these studies was that treatment with 2 mM DHA alone (not as a constituent of a ratio of PUFA) was as effective as the 1:1 PUFA ratio in suppressing EV-induced cytokine release (Fig. 7) and scaffold recellularization (not shown). This result was not surprising, however, as our focus was on the effects of real-world dietary PUFA mixtures, and not on n-3 PUFA treatment alone. Our data also raises the intriguing possibility that n-3 PUFA supplementation may be a less effective anti-inflammatory therapy without a corresponding decrease in n-6 PUFA.

This research is subject to several limitations. We chose to investigate responsiveness to dust-derived EVs in a cell culture model using several lung cell populations. This approach is less relevant than a whole animal model, but establishes the principle that DE-derived EVs have inflammatory activity, and sets a baseline for further experiments using animals. Agricultural workers are likely to be chronically exposed to dust constituents, and our exposure schemes were of a much shorter duration than real-world exposures (2–24 h, or 5 days in scaffold recellularization experiments). However, prolonged dust-exposure studies in mice show an immediate and profound inflammatory response that persists over several weeks^8,34^. Thus, although our design is not a perfect analog for real-world exposure, the use of dust-borne EVs as an inflammatory stimulus is appropriate for these initial in vitro experiments. Although we measured LPS in the purified EV suspension, this may be attributable to the constitutive LPS integral to EVs derived from Gram (−) bacterial membranes, and therefore relevant to studies involving bacterial EVs. We did not however, assess the possibility that the Gram (+) membrane component peptidoglycan (PGN) might have contributed to the inflammatory effects of EV exposure. PGN is known to have inflammatory effects on epithelial cells, although to a modest degree as compared to LPS. Characterization of the PGN-mediated effects of EVs is warranted.

## Conclusion

In summary, a significant share of the inflammatory activity generated by swine CAFO aerosols is attributable to their EV load. The injurious effects of dust-derived EVs in terms of both inappropriate cytokine release, and defects in normal lung repair and regeneration were attenuated following treatment with DHA-enriched PUFA mixtures in three different lung cell populations in vitro. Notably, treatment with DHA-rich PUFA ratios during recovery from EV exposure elicited an amplification of pro-resolution mediators, suggesting in our lung cell in vitro models that n-3-rich PUFA intervention may suppress exaggerated inflammatory responses to EVs carried by an environmental substance. These findings warrant future investigations to assess the translatability of these findings using in vivo models.

## Methods

### Ethics approval

Deidentified human cells and tissues were acquired from the Nebraska Organ Recovery System (NORS) a federally designated non-profit (501(c)(3)) organ recovery organization. All experiments using human cells and tissues were approved by the University of Nebraska Medical Center’s Institutional Review Board in compliance with all State and federal regulations.

### Reagents

Epithelial cell culture medium (BEGM) was obtained from Lonza (Walkersville, MD). DHA (cis-4, 7, 10, 13, 16, 19-docosahexaenoic acid) and arachidonic acid (cis-5,8,11,14-eicosatetraenoic acid) were purchased from MP Biomedicals (Solon, Ohio). Kits for measuring soluble proteins (IL-6, IL-8, TNFα, IL-10) in culture supernates (DuoSet ELISA) were from R&D Systems (Minneapolis, MN). For resolvin D1, kits were from Cayman Chemical (Ann Arbor, MI). ExoQuick-TC exosome isolation reagent was purchased from System Biosciences (Palo Alto, CA). All other reagents were from Fisher Scientific (Pittsburgh, PA) unless otherwise specified.

### CAFO dust extract

Dust was collected from several swine feeding facilities located in northeastern Nebraska and northwestern Iowa housing 500–800 animals as previously described^36^. Briefly, settled dust was collected form horizontal surfaces > 1 m from the floor and suspended in Hanks balanced salt solution (100 mg/mL), incubated for 1 h on a stir plate, centrifuged, and filter sterilized (0.22 µm). This saturated solution was designated 100% dust extract (100% DE) and was stored at − 20 °C for up to 4 months before use. Preparations were virtually free from particulate matter and contained approximately 4 mg/mL total protein. Characterization of the dust extracts has been reported previously^4,5,35^. DE used in experiments was diluted in PBS to a working concentration of 2% or 5%.

### Cell culture

Primary normal human bronchial epithelial cells (HBEC) and lung fibroblasts (HLF) were isolated from deidentified lungs deemed unsuitable for transplant and obtained from tissue repositories at the Nebraska Organ Retrieval System (NORS) in accordance with the guidelines of the University of Nebraska Institutional Review Board. Cell processing details have been published elsewhere^36,61^. HBEC cultures were expanded on collagen-coated dishes in serum-free growth medium containing growth factors (BEGM). HLF were grown in DMEM medium (Cellgrow, Manassas, VA) supplemented with 10% FCS and antibiotics. All cells were maintained in a 37 °C incubator at 5% CO_2_. Cells used in these experiments were passaged fewer than four times. Some experiments utilized the immortalized human bronchial epithelial cell line BEAS-2B (CRL-9609, ATCC Manassas, VA) grown in SF-BEGM medium. Monocyte-derived macrophages (MDM) were generated from THP-1 monocytes (TIB-202, ATCC Manassas, VA) grown in RPMI-1640 medium supplemented with 10% FCS and penicillin/streptomycin and differentiated with 40 ng/mL PMA (phorbol 12-myristate 13-acetate) for 48 h. All cell types grown in serum-containing medium were equilibrated in serum-free formulations before use in experiments. Cells used for experiments involving EVs were maintained in exosome-free serum replacement medium (Gibco KnockOut SR, Thermo Fisher, USA).

### Isolation of DE-derived EVs

Extracellular vesicles (EVs) were precipitated from freshly prepared 100% DE using a volume-excluding polymer-based method (ExoQuick-TC) followed by an additional centrifugation step following a recently published method^62^. Specifically, sterile-filtered DE was centrifuged at 3000×*g* for 15 min at 4 °C to remove cell fragments and protein aggregates. Supernates were then mixed with ExoQuick reagent at a ratio of 1:4 according to the manufacturer’s protocol, and incubated for 12 h at 4 °C. EVs were pelleted at 1500×*g* for 30 min, and again at 1500×*g* for 5 min (4 °C). EV pellets were then resuspended in 12 mL particle-free DPBS and ultra-centrifuged at 100,000×*g* for 80 min at 4 °C using a Beckman-Coulter Optima XPN-90 centrifuge fitted with a SW41Ti swinging bucket rotor. The run was terminated with a deceleration brake setting of 8 (6 min to fully stop). The resulting pellet was resuspended in a volume of sterile DPBS corresponding to one-tenth the original sample volume, stored at 4 °C and used within 48 h for all experiments.

To validate experiments using EVs isolated using the ExoQuick method, EVs were isolated by the more conventional differential centrifugation (DC) method and used to repeat selected experiments. For these experiments, DE prepared as described above was serially centrifuged at 500×*g* for 15 min and 2000×*g* for 15 min, in a Sorvall Legend RT + centrifuge, then at 10,000×*g* for 30 min in a Thermo IEC microfuge at 4 °C, collecting the supernates at each step. The clarified supernates were then passed through an ultrafiltration filter (100K MWT cutoff, Thermo-Pierce) and centrifuged at 3200×*g* for 2 h to further deplete non-EV soluble proteins. The filter retentate (concentrated 30-fold) was then resuspended in particle-free DPBS and centrifuged at 100,000×*g* for 80 min (Beckman-Coulter Optima XPN-90 centrifuge and SW41Ti rotor; brake setting = 8). The resulting pellet was washed in DPBS and recovered with a final 100,000×*g* centrifugation step. Pellets were resuspended in DPBS at a volume corresponding to one-tenth the original sample volume, and stored at 4 °C for not more than 48 h. This method is a standardized approach for high-quality EV isolation^63,64^, and complies with generally recognized guidelines^65^. Results of these validation experiments are included as supplemental material. There was no significant effect of the ExoQuick reagent alone on DE or DE-derived EV-induced modulator release (Supplemental Fig. S3).

### Characterization of DE-derived EVs

Particle size distribution and concentration of EVs isolated from DE samples were analyzed by nanoparticle tracking analysis (Nanosight NS300, Malvern Panalytical, Westborough, MA) using a 532 nm laser and a high sensitivity scientific CMOS camera. Samples were diluted 1:100 in low particle DPBS. Samples were analyzed under constant flow conditions (flow rate = 20 µL/min) at 24 °C. Three 360 s videos were captured for each sample with a camera level of 13. Data was analyzed using NTA 3.1.54 software with a detection threshold of 5 and using automatic adjustment for blur size and jump distance.

### Detergent depletion of EVs

To determine if detergent lysis of DE-derived EVs would modulate their potency, 100% DE was treated with various concentrations of Triton X-100 (10, 100, 150, and 200 mM, corresponding to 0.016, 0.16, 0.24, and 0.32%, respectively) for 2 h at 4 °C. Suspensions were then dialyzed against H_2_O using a 10,000 MWCO membrane (Snakeskin, Thermo Scientific) for 14 h at 4 °C with 6 changes of the dialysate. EVs in the dialysis retentate were then isolated using the differential centrifugation (DC) method (as above), and the resulting pellets were reconstituted at 1/10th the original starting volume, then further diluted to 50 × 10^9^ EVs/mL). EVs treated in this manner were then used to stimulate HBEC cultures for 24 h, supernates collected, and soluble modulators assayed by ELISA. As a control, HBEC growth medium was also treated with Triton, and dialyzed.

### Protein analysis of isolated EVs

A representative sample of EV isolates were analyzed by NTA (nanosight, as above) and the total proteins in the reconstituted pellets were measured for total protein using the Bradford method (BIO-RAD protein assay kit, Hercules, CA). Briefly, protein assay concentrate was diluted 1:4 with ultra-pure molecular biology grade H_2_O. EV samples (10 μL) were mixed with 200 mL diluted reagent in triplicate wells of a 96-well microtiter plate and incubated with shaking for 36 min at room temperature. Total protein values were extrapolated from a standard curve of purified BSA (protease-free fraction V, MP Biomedicals, Irvine, CA). EV abundance was plotted against total protein in the reconstituted EV pellet.

### Endotoxin depletion

Complete 100% DE was depleted of endotoxins by overnight incubation at 4 °C with polymyxin B affinity resin (Detoxigel, Thermo-Pierce, Waltham, MA). Following polymyxin B treatment, resin beads were removed by centrifugation at 1500×*g* (15 min). EVs were isolated, and diluted to two different concentrations (50 × 10^9^/mL or 1.46 × 10^9^/mL) and assayed for endotoxin content (LPS) using a commercial kit following the manufacturer’s instructions (Chromogenic Endotoxin Quant Kit, Thermo-Pierce). The assay measures the chromogenic conversion of endotoxins by the enzymatic action of Limulus amebocyte lysate (LAL). Results were extrapolated from a standard curve of purified *E. coli* endotoxin (provided with the kit) and expressed as EU/mL.

### Genomic analysis of DE-derived EV

DNA was isolated from swine CAFO-derived EV collected from two distinct facilities and prepared as outlined above. DNA was purified from 250 µL of DE-derived EV suspended in phosphate-buffered saline using a QIAamp PowerFecal Pro DNA kit (QIAGEN, Germantown, MD) on a QIAcube automated instrument running the QIAGEN protocol for Powersoil and Powerfecal kits. cDNA libraries were created from dust extract EV DNA using the Nextera XT bead-linked transposome library preparation technique (Illumina Inc., SanDiego, CA). This method simultaneously labels and normalizes library fragments to ~ 350 bp. Whole genome shotgun pyrosequencing (20 million paired (2 × 100 bp) reads) was performed on DNA isolated from DE-derived EV by the UNMC Genomics Core Facility using NGS on the Novaseq 6000 sequence platform (Illumina). Concentrations of all DNA samples used for sequencing were > 5 ng/µL by Nanodrop spectrophotometer (ThermoFisher, Waltham, MA). WGS sequence curation and analyses were performed using python and R, and the following packages: MetaPhlAn v3.0.13, HUMAnN v3.0.1, KneadData v0.11.0, and vegan v2.3-5^66–68^. Briefly, KneadData was used to perform quality control on the raw genomic DNA reads from the metagenomic sequencing run using the standard workflow. After read-level quality control by KneadData, reads were taxonomically classified using MetaPhlAn, and microbial gene families were assigned using HUMAnN (analysis tools acquired on github.com/biobakery). The 50 most abundant bacterial genera, and the top 50 gene families were graphed using the hclust2.py function within MetaPhlAn.

### Negative staining transmission electron microscopy

EVs were imaged by the Electron Microscopy Core Facility at the University of Nebraska Medical Center. Briefly, samples for TEM imaging suspended in HEPES buffered saline were spotted onto formvar/silicon monoxide-coated 200 mesh copper grids (Ted Pella Inc., Redding, CA). Grids were glow discharged for 60 s at 20 µA prior to use. Samples were negatively stained with NanoVan (Nanoprobes, New York, NY) and examined on a FEI *Tecnai G*^*2*^* Spirit* TWIN transmission electron microscope (Hillsboro, OR) operating at an accelerating voltage of 80 kV*.* Images were acquired digitally with an AMT digital imaging system (Woburn, MA). Representative images including diameter measurements are shown in Fig. 1B.

### Treatment of epithelial cells

EVs isolated from DE were interrogated for their ability to induce inflammatory mediator release in a dose response study. BEAS-2B cells were exposed to various concentrations (75 × 10^9^–75 × 10^6^/mL) of DE-derived EVs or complete 5% DE and incubated for 24 h, after which soluble protein release was assessed by ELISA. To further characterize the immunomodulatory effects of DE-derived EVs, complete 100% DE was heated (90 °C, 30 min) or depleted of LPS (excess polymyxin B resin, 12 h @ 4 °C) and EVs were isolated as above. Cells were treated for 24 h with 5% DE, or a 5% DE equivalent concentration of EVs (1.46 × 10^9^ EVs/mL) with and without heat-exposure or LPS ablation, and soluble modulators measured. To determine the contribution of EVs to the inflammatory consequences of DE exposure, HBEC were treated with complete 5% DE, EVs isolated from 5% DE, or the EV-free soluble component of DE (supernates following PEG precipitation, but prior to ultracentrifugation) for 24 h, after which modulators were measured by ELISA. For experiments exploring the effects of treatment with PUFA, HBEC were exposed to DE-derived EVs (50 × 10^9^ EVs/mL) concurrently with mixtures of n-6 PUFA and n-3 PUFA chosen to achieve AA:DHA equimolar ratios of 1:1, 10:1, 20:1 and 50:1. That is, the 1:1 ratio contained 2 µM of each PUFA, and the 50:1 ratio contained 2 µM AA and 40 nM DHA. These concentrations were empirically chosen for their ability to elicit measurable biological effects without cell toxicity and are physiologically relevant (human plasma DHA levels as high as 150 µM have been reported^69^). Cultures were incubated for 24 h and supernates harvested for soluble protein analysis by ELISA.

### Lung scaffold model

Human lung lobes obtained from NORS tissue repositories (as above) were processed using a modification of the methods published by Uhl, et al.^70^ and Gilpin^71^ as previously reported^36,61^. Lung lobes from current smokers, those with a known lung disease, sepsis, lung infection, or obvious lung trauma were excluded. Intact lung lobes were completely decellularized by serial detergent lavage (Triton X-100, sodium deoxycholate) followed by DNase over the course of 7 days and rinsed repeatedly with PBS. Denuded lung lobes were then inflated with low melting point agarose and allowed to solidify at 4 °C. Cylindrical cores were made with a 13 mm biopsy punch and 300 µm sections were made using a vibratome (Precisionary Instruments, Greenville, NC). Scaffold matrix disks (scaffolds) were stored in a 30% ethanol solution at − 20 °C until use. Scaffolds prepared in this manner retain normal lung morphology and ECM components and are devoid of cells^70^. The presence of residual EVs attached to the tissue parenchyma was not evaluated, but if present, were assumed to lack bioactivity due to the stringent preparatory conditions. For experiments, each scaffold was equilibrated in culture medium before being seeded with 1 × 10^5^ HBEC and incubated for 4 h to allow attachment. Scaffold cultures were moved to fresh culture dishes and treated with 50 × 10^9^ DE-derived EVs/mL for 5d. Following this initial exposure phase, scaffold cultures were rinsed, and treated with three mixtures of n-6 and n-3 PUFA (AA:DHA equimolar ratios of 1:1, 20:1 and 50:1, as described above, or with 2 mM DHA alone) for an additional 3d. Cultures were re-fed with treatments every second day. At the conclusion of the recellularization period, scaffolds were rinsed, removed to fresh dishes and the number of adherent cells was assessed using a cell proliferation kit (Vybrant MTT proliferation assay, Thermo Fisher, Waltham, MA) by interpolating against a standard curve of labeled cells in suspension.

### Wound repair assays

Primary HBEC and HLF were grown to confluency on collagen-coated tissue culture cluster plates. HLF cultures were serum-starved for 24 h before the experiments were begun. Circular wounds were made in the center of each well using a modified inoculation loop (0.5 mm dia.), wells were rinsed once to remove cell debris and photographed using a low-power objective (initial wound area, T_0_). Wounded monolayers were then treated with DE-derived EVs (50 × 10^9^/mL), EVs + PUFA ratios, or medium alone. After 6, 18 and 24 h, the wells were photographed and wound area was quantified using NIH ImageJ software and compared to the initial wound area, with each well serving as its own baseline control. Wound repair was reported as percent of initial wound area at T_0_.

### Soluble protein analysis

Inflammatory mediators released by in vitro cell cultures were measured by ELISA using commercially available kits following manufacturer’s instructions. Human IL-6, IL-8, TNFα, AREG, IL-10 (R&D Systems DuoSet ELISA development kits) and RvD1 (Cayman Chemical) were measured in cell-free supernates. Limits of detectability were 9.4, 31.2, 12.5, 15.6, 12, and 3.3 pg/mL, respectively, for the soluble proteins listed above).

### Statistical methods

Graphical representations and statistical analyses were performed using Graphpad Prism software (San Diego, CA). Boxplots represent medians of all data ± quartiles with whiskers encompassing the entire range. Calculations of significance for most experiments were performed using one-way ANOVA with Tukey’s post-hoc multiple comparisons test. For some experiments, pairwise comparisons were made using Student’s *t* test (e.g., where two treatments were compared at a given time point). For all analyses, a p-value of less than 0.05 was considered significant.

## Supplementary Information

Supplementary Figure S1.

Supplementary Figure S2.

Supplementary Figure S3.

## Data Availability

Sequencing data is deposited in the National Center for Biotechnology Information Sequence Read Archive (BioProject ID: PRJNA846770). The data are under embargo from the site: “This Sequence Read Archive (SRA) submission will be released on 2023-07-01 or upon publication, whichever is first.” After the paper is published, it will be released. An active link is provided for the editor, and reviewer, if appropriate, to verify that the data are uploaded. https://dataview.ncbi.nlm.nih.gov/object/PRJNA846770?reviewer=66li4vdda3l8kafumhntgkij11. All additional data, which support the findings of this study, are available within the paper or from the corresponding author upon request.

## Abbreviations

CAFO: Concentrated animal feeding operation
DE: Dust extract
HBEC: Primary human bronchial epithelial cells
HLF: Primary human lung fibroblasts
MDM: Monocyte-derived macrophages
PUFA: Polyunsaturated fatty acids
DHA: Docosahexaenoic acid (22:6(n-3))
AA: Arachidonic acid (20:4(n-6))
EVs: Extracellular vesicles
LPS: Lipopolysaccharide
RvD1: Resolvin D1
PEG: Polyethylene glycol
