# Supplementary material for: Agricultural dust derived bacterial extracellular vesicle mediated inflammation is attenuated by DHA
Source: Sci Rep. 2023 Feb 16;13:2767. doi: 10.1038/s41598-023-29781-9 (PMC9933036; doi:10.1038/s41598-023-29781-9)
Supplement: Supplementary file 1 — Supplementary Figure S1. [file 41598_2023_29781_MOESM1_ESM.pdf]

Supplemental Figure S1

Validation of EV dose response using differential centrifugation as the EV isolation method.

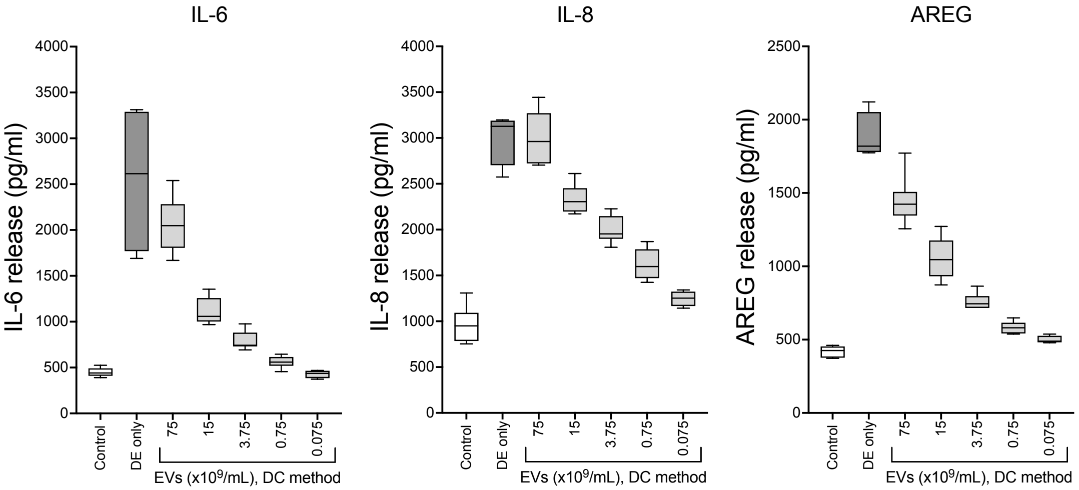

S1. EVs were isolated using a traditional differential centrifugation technique: DE prepared as described in the Methods section was serially centrifuged at 500 x g for 15 min. and 2000 x g for 15 min., in a Sorvall Legend RT+ centrifuge, then at 10,000 x g for 30 min. in a Thermo IEC microfuge at 4ºC, collecting the supernates at each step. The clarified supernates were then passed through a size-exclusion filter (100K MWCO, Thermo-Pierce) and centrifuged at 3200 x g for 2h to further deplete non-EV soluble proteins. The filter retentate (concentrated 30-fold) was then resuspended in particle-free DPBS and centrifuged at 100,000 x g for 80 min. (Beckman-Coulter Optima XPN-90 centrifuge and SW41Ti rotor; brake setting = 8). The resulting pellet was washed in DPBS and recovered with a final 100,000 x g centrifugation step. Pellets were resuspended in DPBS at a volume corresponding to one-tenth the original sample volume and stored at 4ºC for not more than 48h. Primary human HBEC were challenged with EVs at concentrations ranging from 75 x 10^9 to 75 x 10^6 particles/mL for 24h, and soluble protein release measured by ELISA. Eight technical replicates per condition. See manuscript Figure 3A for a comparison of the results using the PEG-based precipitation method.
