# Supplementary material for: Agricultural dust derived bacterial extracellular vesicle mediated inflammation is attenuated by DHA
Source: Sci Rep. 2023 Feb 16;13:2767. doi: 10.1038/s41598-023-29781-9 (PMC9933036; doi:10.1038/s41598-023-29781-9)
Supplement: Supplementary file 2 — Supplementary Figure S2. [file 41598_2023_29781_MOESM2_ESM.pdf]

Supplemental Figure S2

Validation of PUFA ratio response using EVs isolated by differential centrifugation

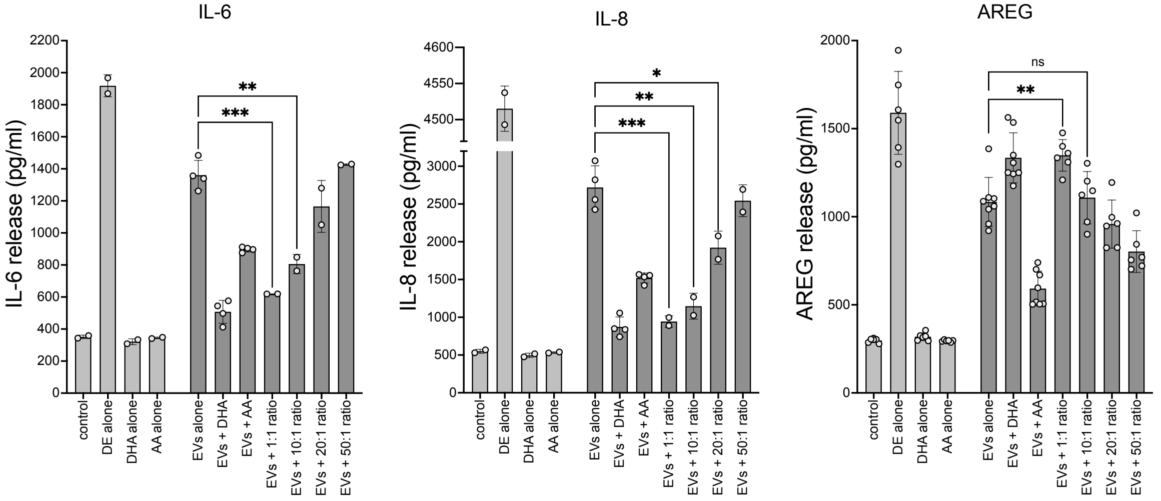

S4. Effect of AA to DHA PUFA ratios on EV-induced modulator release from primary HBEC using EVs isolated using the size-exclusion filter + differential centrifugation (DC) technique. Refer to the caption for Supplemental S1 and amended Methods section in the manuscript for isolation method. HBEC were challenged with EVs isolated using the DC method (50×10^9/mL) or with complete 5% DE, or 2 μM DHA, or 2 μM AA alone for 2h, then EVs were removed, and cultures were allowed to recover in the presence of four different PUFA ratios for 22h. Release of EV-mediated proinflammatory cytokines IL-6 and IL-8 was attenuated, and pro-repair AREG was modestly augmented by treatment with the DHA-rich (1:1) PUFA ratio and by DHA alone during the recovery phase. Results of experiments using EVs isolated by differential centrifugation + filtration were similar to the results shown in Figure 7 of the manuscript; DC-isolated EVs stimulated modulator release, and DHA-rich PUFA ratios attenuated this effect.
