# Supplementary material for: Agricultural dust derived bacterial extracellular vesicle mediated inflammation is attenuated by DHA
Source: Sci Rep. 2023 Feb 16;13:2767. doi: 10.1038/s41598-023-29781-9 (PMC9933036; doi:10.1038/s41598-023-29781-9)
Supplement: Supplementary file 3 — Supplementary Figure S3. [file 41598_2023_29781_MOESM3_ESM.pdf]

Supplemental Figure S3

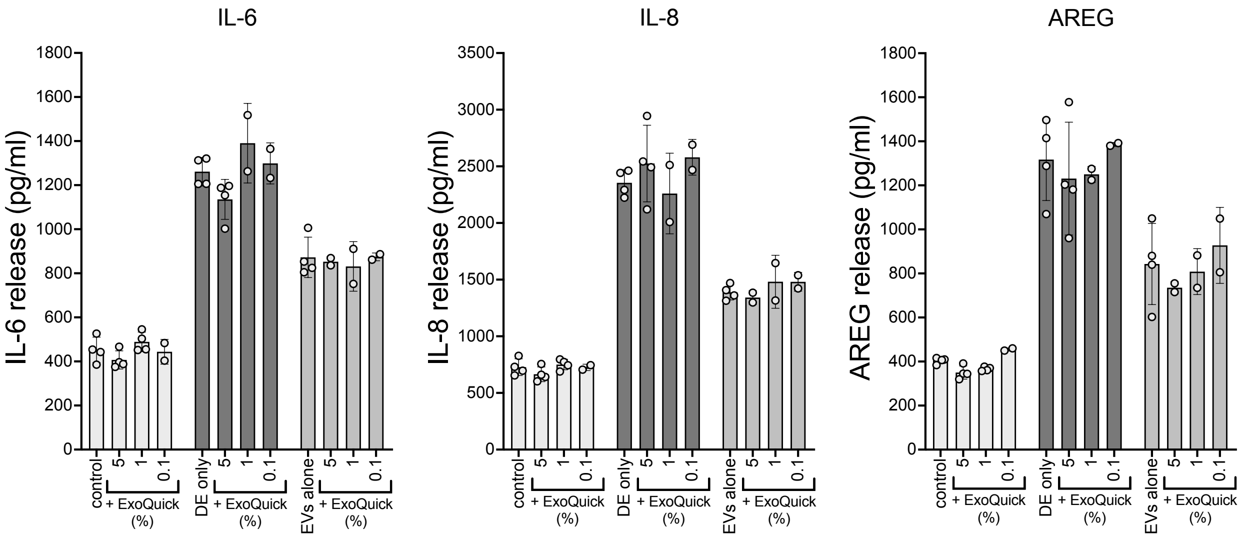

S3. Effects of ExoQuick reagent on the release of cytokines from HBEC cultures. Control medium, complete DE, and DE-derived EVs (EVs alone) isolated using differential centrifugation as above, (S1) and resuspended at 50 x 10^9/mL) were incubated with diluted ExoQuick reagent (5%, 1%, 0.1%) for 1h. ExoQuick-spiked solutions were then used to treat HBEC cultures for 24h. The ExoQuick reagent (even at high concentrations) had negligible effects on modulator release for all treatments.
